# Self-congruity and functional congruity drive positive word-of-mouth in food tourism through moderating effects of emotional experiences

**DOI:** 10.1038/s41598-025-94046-6

**Published:** 2025-03-27

**Authors:** Danping Liu, Miaoxuan Wu, Tingting Zhu, Hedan Fang, Di Hu

**Affiliations:** 1 School of Management, Xihua University, Chengdu, 610039 China; 2 Research Institute of International Economics and Management, Xihua University, Chengdu, 610039 China; 3 School of Logistics and Management Engineering, Yunnan University of Finance and Economics, Yunnan, 650300 China; 4 School of Business, Anhui University of Technology, Ma’anshan, 243032 China

**Keywords:** Self-congruity, Functional congruity, Tourists’ emotional experiences, Destination personality, Positive word of mouth, Psychology, Psychology and behaviour

## Abstract

This research aims to develop an expanded model integrating destination personality, tourists’ emotional experiences, and positive word of mouth within the frameworks of self-congruity and functional congruity. Using Partial Least Squares Structural Equation Modeling (PLS-SEM), we analyzed data from 357 tourists who visited the UNESCO-designated City of Food in China. Our findings indicate that destination personality, self-congruity, functional congruity, and tourists’ emotional experiences positively influence positive word of mouth. Notably, tourists’ emotional experiences were found to moderate the relationship between self-congruity and positive word of mouth. This research enhances the existing literature by addressing a significant gap and introducing a comprehensive conceptual model that is empirically validated. The study provides actionable insights for optimizing food tourism marketing strategies, offering theoretical and practical implications for practitioners and managers, thus making a significant contribution to the discourse on food tourism.

**Supplementary Information:**

The online version contains supplementary material available at 10.1038/s41598-025-94046-6.

## Introduction

Food tourism has emerged as a crucial element of destination marketing, generating significant economic impact by attracting tourists seeking unique culinary experiences^1,2^. This growing trend highlights the increasing role of gastronomy in tourism, with an expanding number of travelers motivated to explore and invest in novel dining opportunities^3^. Defined by the pursuit of distinctive and memorable food and beverage encounters, food tourism not only facilitates the discovery of indigenous cuisines but also fosters an understanding of the reciprocal relationship between tourists and their destinations^4^. Although numerous empirical studies have examined the influence of destination personality, self-congruity, and functional congruity on consumer behavior, such as willingness to visit, satisfaction, and word of mouth recommendations^5–9^, few have incorporated tourists’ emotional experiences into this framework^10,11^. This omission is notable given the distinctiveness of gastronomic tourism, where tourists often seek memorable, culturally rich experiences that go beyond typical tourism activities^12^. Thus, a key question arises: How do tourists’ emotional experiences influence the relationships between self-congruity and functional congruity in shaping word of mouth behaviors? The aim of this study is to develop and empirically test a theoretical framework that elucidates the interconnections among destination personality, self-congruity, functional congruity, tourists’ emotional experiences, and their propensity for positive word of mouth within the food tourism sector. Specifically, this research explores the combined effects of self-congruity and functional congruity on tourists’ intentions, with a particular focus on how emotional experiences—such as feelings of joy, love, and positive surprise—mediate the link between congruity perceptions and favorable word of mouth outcomes.

Recent advancements in tourism research have expanded the theoretical framework for predicting travel behavior by integrating concepts such as destination image, place attachment, cultural influences, and memory experiences^13–16^. Despite this progress, the inclusion of tourists’ emotional experiences within predictive models remains notably underexplored, with the exception of a conceptual analysis by Yang^17^. Empirical studies investigating the moderating role of emotional experiences in tourism decision-making are scarce^10,11,17^. Moreover, while food tourism has garnered increasing scholarly attention, the emotional experiences of gastronomic tourists remain underrepresented in the literature^18^. Positive emotional experiences, which tourists seek to enhance their journeys, are particularly significant, as travelers often expect enjoyable, memorable encounters while striving to avoid negative emotions^19^. These experiences are critical, aligning with Trauer’s intimacy theory^4^, which suggests that destinations evoking positive emotions are perceived as more homelike by tourists. Empirical evidence further highlights the impact of emotional states on satisfaction, loyalty, purchasing behavior, and engagement with leisure services^20–23^. However, the extent to which emotional experiences influence behavioral intentions, particularly in the context of food tourism and their role in word of mouth promotion, remains unclear. Given the pivotal role of emotional intensity in shaping word of mouth dynamics, exploring the emotional drivers behind such endorsements among food tourists is an essential area for future research.

The emotional experiences of tourists play a pivotal role in shaping tourism outcomes^22–24^. Before a trip, potential tourists often engage in reflective processes, recalling past experiences to inform their destination choices. This emotive-driven decision-making mechanism, identified as the ‘drug motive’, significantly influences future behavioral intentions toward travel^15^. Throughout the journey, emotional engagement continues to act as a catalyst for motivational activation, further shaping travel behaviors^25^. Post-tour, a heightened level of active engagement with the destination often leads tourists to advocate for the location to their networks^26^. This sequence underscores the critical role of emotional experiences not just in destination perception but also in the broader behavioral patterns of travelers^10,27^. Within the realm of tourism, emotional experiences are identified as a core component, a principle that extends to food tourism as well. Engaging deeply with gastronomic experiences elicits distinct emotions among food tourists, which in turn influences their behavior and intentions^28^. Furthermore, the exploration of various model structures within the gastronomic context promises to enhance our understanding of food tourist behavior, pointing toward a rich avenue for future research^4^.

In the last twenty years, special interest tourism has established itself as a considerable segment within the tourism industry, serving as a profitable channel for destinations that provide a diverse range of products and acting as a crucial support for locales focusing on a singular specialty^29,30^. Derrett^31^conceptualizes special interest tourism as customized leisure and recreational activities catered to the unique preferences of individuals, encompassing diverse forms such as yoga, red (revolutionary history), religious, senior, and cultural tourism. Among these, food tourism emerges as a quintessential example, serving not only as a pivotal component of travel but also as a crucial medium for identity construction in postmodern societies^32^. This trend underscores a growing predilection among travelers to fulfill higher-level needs via culinary exploration, positioning food tourism as a key catalyst for the sophisticated evolution of China’s tourism industry^33^.

Theories of self-congruity and functional congruity have been instrumental in predicting key behavioral intentions, such as word of mouth, satisfaction, customer loyalty, and revisit intentions^34–37^. Pioneering work by Sirgy and Su^7^laid the foundation for further research in tourism, demonstrating the critical role of both personal and functional congruence in forecasting visitor behavior^5,6,34,38–40^. In the context of tourism, self-congruity refers to the alignment between a traveler’s self-image and the characteristics of a destination, thereby enhancing the tourist’s emotional connection to the place^17^. In contrast, functional congruity pertains to how well a destination’s attributes meet the expectations and needs of travelers^41^. These concepts have also been extensively applied in studies exploring the relationship between branding and congruity^42,43^. Destinations, much like brands, are selected by tourists based on their alignment with personal values and desires. A growing body of literature has affirmed the strong relationship between self-congruity and functional congruity in shaping destination personality and image^17,35,44,45^. Accordingly, self-congruity and functional congruity serve as mechanisms linking destinations to tourists’ behaviors, explaining why tourists are more likely to choose destinations that resonate with their self-concept. Furthermore, the better the match between a tourist’s self-concept and a destination’s personality, the more likely it is to result in positive behavioral intentions. Food tourism destinations share similarities with traditional tourist destinations, suggesting that this theoretical framework can be extended to gastronomic tourism. Beyond merely offering memorable dining experiences, food tourism destinations cultivate emotional and cognitive bonds between tourists and the place^33,46^. These emotional connections underscore the profound relationship between travelers and their chosen destinations. Understanding broader patterns of tourist behavior requires a focus on the role of emotional experiences in shaping perceptions of and interactions with tourist locations^10,27^. While Yang et al.^17^ proposed a theoretical framework incorporating emotional experiences, empirical evidence on how such experiences influence congruence theory’s impact on tourist behavior remains limited. Thus, the theories of self-congruity and functional congruity provide a robust theoretical lens for exploring the role of emotional experiences in food tourism.

This study offers significant contributions to both theoretical and applied domains. From a theoretical perspective, it introduces an innovative moderation role for tourists’ emotional experiences in the relationship between self-congruity, functional congruity, and tourist behavior. By examining the complex interactions among destination personality, congruence constructs, emotional experiences, and promotional behaviors within the context of food tourism, this research enriches the existing theoretical framework. Furthermore, it highlights the critical role of functional congruity in destination matching, extending the application of functional congruity theory. From a practical standpoint, the insights derived from the proposed framework provide valuable guidance for tourism practitioners and policymakers, particularly those focused on food tourism. These insights could serve as a strategic tool for more effectively attracting and engaging targeted tourist segments.

## Theoretical background and research hypotheses

### Self-congruity (SC)

Self-congruity refers to the alignment between an individual’s self-concept and the symbolic representation of a product, brand, or retailer, distinguishing it from traditional functional perspectives. This concept emerges when an individual perceives a strong resonance between a contextual interaction—whether with a product, medium, or other stimuli—and their ideal self, encompassing their interests, values, and needs. Such alignment fosters self-referential motivation, promoting authenticity and increasing self-involvement^45^. As a result, the degree to which a consumer’s perception of a product’s image aligns with their self-concept significantly influences their preference for the product and purchasing decisions^46^. Post-purchase, self-congruity continues to impact key outcomes, with evidence showing its positive effects on customer loyalty^47^ and satisfaction^48^.

Chon^49^ was among the first to apply self-congruity theory to tourism, proposing that self-congruity occurs when there is alignment between an individual’s self-perception and their perception of a destination. This framework suggests that travel choices are shaped by personal perceptions and lifestyles^50^, with individuals favoring destinations that resonate with their ideal self-concept^17^. The degree of congruity between a tourist and a destination significantly affects their behavioral intentions, with greater congruity leading to stronger intentions^34^. Subsequent research has further elucidated the impact of self-congruity on various dimensions of tourist behavior, revealing positive correlations with behavioral intentions^34,44^, tourist commitment^51^, and brand loyalty to destinations^52^. In the context of food tourism, self-congruity has been explored as a precursor to tourist behavior^53,54^. However, empirical studies that apply self-congruity to develop more flexible and comprehensive structural equation models remain limited.

### Functional congruity (FC)

In marketing research, Image Congruity Theory encompasses two key components: self-congruity and functional congruity, as outlined by Sirgy et al.^34^. Both self-congruity and functional congruity serve as antecedents to word of mouth^55–57^, and their integration into research models provides a comprehensive understanding of consumer behavior^58,59^. However, some scholars have suggested that functional congruity exerts a weaker influence on consumers compared to self-congruity^40,60^.

Self-congruity refers to the cognitive alignment between an individual’s self-perception and the image of a product’s brand, capturing the emotional and psychological dimensions of congruence. In contrast, functional congruity addresses the rational aspect, focusing on the utilitarian qualities of a product, such as price, comfort, safety, and convenience. This dimension plays a critical role in consumers’ post-purchase evaluations and behaviors, underscoring the importance of functional utility in shaping consumer satisfaction^60^. In the context of tourism, functional congruity pertains to the extent to which a destination’s practical features align with tourists’ expectations, as articulated by Chon and Olsen^45^. It involves a cognitive evaluation of how well a destination fulfills tourists’ practical needs^61^, with higher levels of functional congruity reflecting a closer match between the desired benefits of tourists and the destination’s offerings, thus significantly influencing travel decisions and behaviors.

Functional congruity has been shown to influence travelers’ behavioral intentions in several studies^36,62^. However, its application within culinary tourism remains underexplored^63^. In the context of national brand food purchase intentions, Shin^55^ suggested that self-congruity assessments may occur alongside evaluations of functional benefits, indicating the potential relevance of functional congruity in food tourism. Functional congruity in this context refers to tourists’ satisfaction with the practical aspects of their experience, such as food and beverage quality, accommodation comfort, visitor congestion, and the variety and originality of activities^64^. Bosnjak et al.^65^ further expanded this concept by incorporating a destination’s functional image, which includes amenities, accessibility, fine dining, recreational opportunities, cultural attractions, and overall environmental quality. This extension highlights the importance of examining functional congruity in food tourism and advocates for a more nuanced analysis of both functional and self-congruity to comprehensively understand their combined influence on tourist experiences. In sum, this underscores the motivations for exploring functional congruity within the food tourism domain.

### Tourists’ emotional experiences

The rapidly growing field of mass tourism has increasingly highlighted the tourism experience as a central element of travel, garnering substantial scholarly attention. Otto and Ritchie^66^ defined the travel experience as a personal psychological state shaped by interactions with services. Mehrabian and Russell^67^ argued that travel experiences provide a meaningful way to address real-life challenges, with travelers seeking authenticity. This perspective reflects a broader scholarly consensus that tourism experiences offer significant value to both individuals and society. Furthermore, Mayo et al.^68^ emphasized the profound influence of both internal psychological and external social factors on tourism decision-making processes. A key aspect of these discussions is the emotional experience of tourism, which Aho^69^ identified as a critical component of the tourism journey. This highlights the necessity of adopting an emotional framework to fully understand the complexities of the tourism experience.

Emotions, defined as intense affective states linked to specific referents and characterized by episodic feelings, drive distinct behavioral responses^70^. They arise from an individual’s psychological interpretation and cognitive evaluation of external events or situations. In the tourism sector, both positive and negative emotions significantly shape tourists’ perceptions, influencing their assessments of experiences and destination choices^71,72^. Research by Hosany et al.^73^ and Prayag et al.^19^ has further highlighted the substantial impact of specific emotions on tourists’ perceptions of destinations and their subsequent behavioral intentions. The exploration of emotional experiences and behaviors provides valuable insights into the psychological foundations of tourist actions, suggesting a more nuanced understanding of the tourism experience^74^. Huang et al.^75^ and Kim et al.^76^ have emphasized the importance of examining the psychological mechanisms behind tourist behavior to enhance our understanding of tourism experiences. Despite a growing body of literature on emotional experiences in tourism, studies specifically addressing emotions in the context of tourist destinations remain limited^22^.

### Destination personality and self-congruity

Destination personality is an extension of brand personality, where the destination is viewed as a product or brand. Brand personality is product-specific, while destination personality attributes human-like traits to destinations. Aggarwal et al.^77^ demonstrated that consumers are attracted to brands exhibiting traits, intentions, or emotions akin to human qualities, aligning with the human tendency to form social bonds with their environment^78^. This inclination towards anthropomorphizing—attributing human traits to inanimate objects, organisms, goods, and brands—is well-documented^79,80^. In tourism, researchers suggest that destinations, as the quintessential products or brands of countries^81^, possess attributes similar to human personality, collectively known as destination personality^82,83^. Moons et al.^84^ further elaborate that destination personalities encompass both tangible and intangible attributes, fulfilling various symbolic and utilitarian functions. Distinctive and appealing destination personalities, such as Europe’s traditional sophistication, Barcelona and New York’s vibrancy, Paris’ romantic allure, Africa’s untamed nature, and Korea’s authenticity, significantly influence consumer perceptions, choices, and emotions^35,84–86^, thereby enhancing the destination’s image and brand equity^34,82^.

Self-congruity and destination personality are pivotal cognitive constructs in destination marketing88, where self-congruity measures the congruity between a tourist’s self-perception and a destination’s perceived character^87,88^. Studies from a variety of fields have highlighted that consumers are drawn to and more inclined to engage with brands that align with their self-perception^89,90^. In tourism research, theoretical models exploring the relationship between destination personality and self-congruity have been empirically validated, consistently demonstrating destination personality’s significant impact on enhancing self-congruity^17,36,91^. This dynamic is particularly evident in food tourism, where the distinct personality of a destination and its alignment with food tourists’ self-perception markedly shapes their experience. Based on these findings, we suggest the following hypothesis:

H1: Destination personality exerts a positive influence on self-congruity.

### Destination personality and functional congruity

Destinations are recognized not solely for their symbolic value but also for their functional utility^40^, encompassing amenities, services, value, accessibility, and information—factors that meet essential travel requirements and play a role in creating a destination’s practical image^7,27^. These aspects have a notable effect on tourist actions. Within the tourism literature, Su and Reynolds^92^ have shown that certain traits of destination personality, such as vibrancy and capability, are critical in shaping tourists’ perceptions of functional congruity, particularly in hotel contexts, underscoring the influence of destination personality on functional congruity perceptions. Nonetheless, Sop^36^ contended that the significance of functional congruity in tourism research has been undervalued. Subsequently, Zhou et al.^62^ established that tourists’ cognitive assessments of a destination’s ability to meet their utilitarian needs directly affect their behavioral intentions. Specifically, in food tourism, the uniqueness of local cuisine not only accentuates a destination’s personality but also provides essential functions like local food offerings, transportation, and convenience services, illustrating a symbiotic relationship between consumer expectations and destination offerings. This leads us to posit that a more pronounced destination personality enhances functional congruity, thereby better aligning with consumer expectations. Thus, we hypothesize:

H2: Destination personality exerts a positive influence on functional congruity.

### Self-congruity and functional congruity

Self-congruity and functional congruity jointly elucidate consumer behaviors, offering complementary perspectives^25,85,86^. Within destination marketing, both self-congruity and functional congruity have been independently leveraged to forecast behavioral intentions^56^. Self-congruity shapes tourists’ initial perceptions of a destination, influencing how subsequent information is processed^25^. Notably, self-congruity extends its impact on behavior through an indirect pathway via functional congruity, a phenomenon described as the ‘self-congruity bias’^40^. This bias suggests that heightened self-congruity results in more favorable assessments of a destination’s functional attributes^37,93^, with pioneering evidence provided by Hung et al.^94,95^. Subsequent research has corroborated the effect of self-congruity on the functional perceptions of hotels and destinations^40,96^. Within the scope of food tourism, it is proposed that the closer the match between tourists’ self-perception and the destination, the more enhanced the experience and the resulting practical advantages gained. This premise forms the basis of the following hypothesis:

H3: Self-congruity exerts a positive influence on functional congruity.

### Self-congruity, functional congruity, and positive word of mouth

Word of mouth is characterized as the behavior where customers express their attitudes toward a brand, product, or service^6^, typically emerging from unofficial sources. Research indicates that when tourists perceive a strong alignment between their self-perception and a destination, they are more likely to engage in positive word of mouth behaviors about that destination. This phenomenon arises from the tourist’s self-congruity with the destination, leading to positive behaviors^43,97–99^. Šegota et al.^6^ demonstrated that ideal social self-congruity significantly enhances many-to-many word of mouth, suggesting that the more an individual desires to be associated with a place, the more likely they are to endorse or defend it in social settings. In food tourism, a higher alignment between a traveler’s self-perception and the food tourism destination strengthens their destination preference, influencing their attitudes and behavioral intentions. Positive congruity increases tourists’ propensity to appraise the destination favorably. Based on these insights, we propose the following hypothesis:

H4: Self-congruity exerts a positive influence on positive word of mouth.

Sirgy et al.^99^ conceptualize functional congruity as stemming from the evaluation of a product’s or brand’s performance against the consumer’s ideal expectations. This evaluation addresses consumer inquiries regarding the product’s ability to fulfill their needs and achieve their desires through its utilitarian attributes^100,101^. Extant research underscores functional congruity’s positive impact on pivotal tourism behaviors, including satisfaction, loyalty, and destination selection^7,44,65^. Given that positive word of mouth is a critical behavioral outcome, it is posited that a higher concordance between the actual performance of a destination and tourists’ expectations would correspondingly augment positive word of mouth. Specifically, in the context of food tourism, engagement in activities like sampling local cuisines, experiencing unique dining services, and immersing in the local food culture enhances tourists’ satisfaction with the functional benefits sought, thereby aligning the food tourism destination more closely with tourists’ functional needs. A greater degree of such alignment is likely to foster identification with the destination, subsequently affecting their attitudes and behavioral intentions. Based on this understanding, we propose the hypothesis:

H5: Functional congruity exerts a positive influence on positive word of mouth.

### Moderating role of tourists’ emotional experiences

Self-congruity may not have a direct effect on tourist intentions^102^, suggesting that moderating variables must be considered when assessing the influence of congruity on destination behaviors^35^. According to the broaden-and-build hypothesis^103,104^, positive emotional experiences expand individuals’ temporary cognitive and behavioral repertoires, fostering the accumulation of long-term personal resources, including social, psychological, intellectual, and physical assets. Travelers’ emotional experiences, which are transient states triggered during travel, can facilitate personal engagement with activities and environments^105^. Emotionally aroused tourists are more likely to share narratives that align with their self-identity, as such emotional states enhance their recollection of experiences^44^.

This moderating mechanism is further supported by empirical research. Su et al.^91^ found that emotional experiences regulate narrative readiness across a range of tasks. Similarly, intentions to revisit and recommend have been closely linked to emotions such as joy, love, and pleasant surprise^106,107^. Kim and Ritchie^76^ demonstrated that the relationship between behavioral intentions and self-concept is mediated by emotionally charged experiences, including astonishment and nostalgia. These findings suggest that emotions act as a gatekeeper in transforming congruity into advocacy. One study further revealed that emotions bridge the ‘intent-action’ gap, showing that travelers with high self-congruity exhibited significant positive word of mouth intentions only when their emotional engagement surpassed a certain threshold^108^. Similarly, Boksberger et al.^66^ highlighted that emotional fulfillment mediates the impact of self-congruity on loyalty behaviors, such as recommendations. This aligns with cognitive appraisal theory^109^, which posits that emotions shape post-experience evaluations by filtering cognitive perceptions. Destination experiences are key in eliciting emotions and crafting memorable encounters for tourists^100,110,111^. In the context of food tourism, experiences such as savoring indigenous dishes or enjoying exceptional service can trigger emotions of joy, love, and positive surprise, thereby influencing the effect of self-congruity on behavioral outcomes. It is hypothesized that tourists with higher self-congruity are more likely to promote destinations actively when their emotional experiences are notably positive. This leads to the following proposition:

H6: Tourists’ emotional experiences (joy, love, positive surprise) moderate the relationship between self-congruity and positive word of mouth.

Functional congruity refers to the perceived alignment between the practical needs of travelers and the physical attributes of a destination^112^. In tourism, functional congruity has been identified as a key precursor to positive word of mouth. However, negative word of mouth can still arise if travelers’ functional requirements are met but the emotional experience remains lacking. The strength of this relationship is contingent upon several factors, with post-experience evaluations largely driven by emotions. When functional congruity is high—such as in destinations offering seamless transportation or quality accommodations—tourists’ emotional experiences enhance their pleasure and their likelihood of recommending the destination^113^. This aligns with the broaden-and-build theory, which posits that positive emotions increase cognitive flexibility, making functional advantages more salient in memory and narrative^104^. Han et al.^113^ found that even highly functional destinations, such as those with efficient transport systems, fail to inspire positive word of mouth unless tourists experience emotions like delight or gratitude. Similarly, Prayag et al.^104^ demonstrated that the impact of functional congruity on positive word of mouth is mediated by emotional engagement, particularly in historical tourism contexts, where advocacy is driven not only by logistical efficiency but also by emotional resonance. To foster positive word of mouth, functional adequacy—such as safety and affordability—must evoke emotions like joy or trust, as purely rational assessments lack the motivational power of emotional states^10,114^. In this regard, the emotional experiences of tourists play a critical mediating role in transforming utilitarian evaluations into passionate advocacy.

Within the domain of food tourism, the depth of the culinary experience and the convenience of facilities provided by a destination significantly enhance the perceived functional benefits of consumers. This heightened perception triggers emotional responses and specific behavioral outcomes, as elucidated by Cohen and Areni^70^. Namely, the emotional experiences tourists undergo at a food destination serve as a moderating factor for certain behaviors, such as word of mouth. Travelers are more likely to evaluate a destination positively when their emotional experiences correspond with the expected advantages^115^. Drawing from these observations, we suggest the following hypothesis:

H7: Tourists’ emotional experiences (joy, love, positive surprise) moderate the relationship between functional congruity and positive word of mouth.

**Fig. 1:**
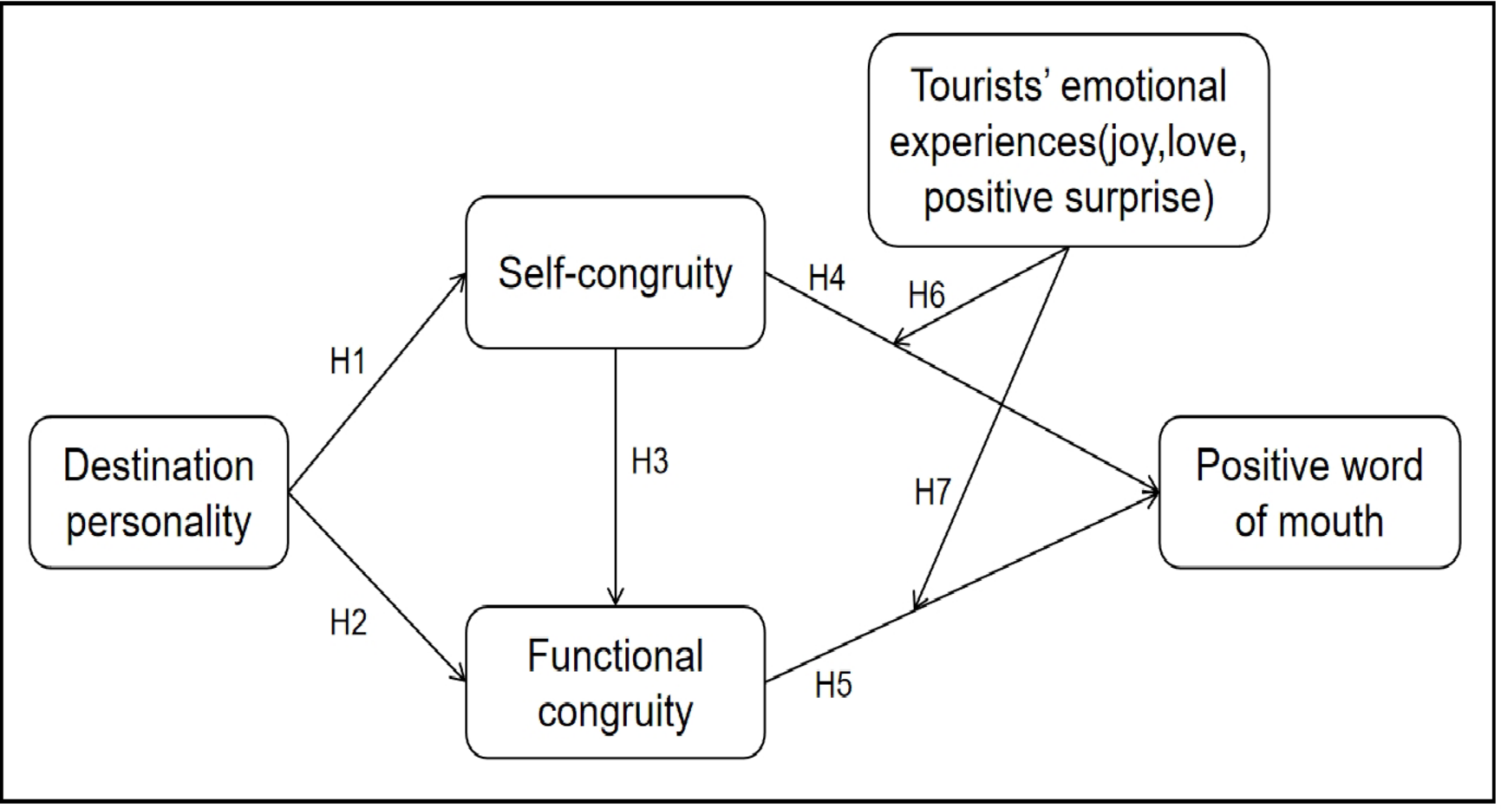
Conceptual model.

The conceptual model depicted in Fig. 1 illustrates how destination personality shapes both self- and functional congruity, which, in turn, influences tourists’ emotional experiences and their subsequent propensity for positive word of mouth.

## Research method

### Study setting and sample size

As of September 2021, UNESCO has designated nine cities and regions worldwide with the prestigious “City of Food” title, five of which are located in China: Chengdu, Shunde, Macao, Yangzhou, and Huai’an. This study focuses exclusively on these five Chinese destinations, which are renowned globally for their culinary culture, preparation, and experiences, and attract a high volume of both domestic and international tourists. Given their prominence, these destinations provide an ideal context for data collection. Consequently, other UNESCO “City of Food” locations outside China are not considered in this study. The destination personality measure in the questionnaire was adapted from Kumar^115^, consisting of 23 items across three sub-dimensions. The self-congruity and functional congruity metrics were derived from the frameworks of Šegota et al.^6^ and Usakli et al.^40^. The measurement of travelers’ emotional experiences was expanded to include four sub-dimensions, building upon the work of Hosany and Prayag^11^ and Sharma and Nayak^10^. Finally, positive word of mouth was assessed using four items based on the research of Breitsohl and Garrod^116^ and Thompson et al.^117^.

To empirically evaluate the proposed research model, we targeted non-local tourists who had visited any of the five designated cities. To ensure informed participation, the lead researcher provided a brief overview of the study’s purpose and ethical considerations at the outset of the questionnaire. The survey, which took approximately 15 min to complete, emphasized respondents’ anonymity and confidentiality. The questionnaire was distributed via the WeChat platform using purposive sampling. Eligibility was determined through a screening question: Which of the following cities or regions have you visited? Respondents were instructed to exclude their current place of residence, ensuring that only non-local tourists who had visited at least one of the five cities could proceed with the survey. Otherwise, the survey was terminated. Following the recommendations of Hair et al.^118,119^, which suggest a minimum sample size of 200–400 for structural equation modeling (PLS-SEM) to detect a minimum effect at specified power and significance levels, our data collection occurred online between August and September 2022. This process yielded 452 completed questionnaires, satisfying the minimum sample size for PLS-SEM. Moreover, similar sample sizes have been employed in empirical studies of tourist behavior^120–122^. To ensure data validity, we followed Curran’s^123^ approach, refining the dataset by excluding outliers, rapid responses, and patterned answers. After these adjustments, a final sample of 357 valid questionnaires remained for analysis.

### Data analysis

The research employed Partial Least Squares Structural Equation Modeling (PLS-SEM) for data analysis, utilizing SmartPLS 3.2.9 software. As noted by Hair et al.^124^, second-order constructs can be modeled to reduce the number of hypotheses and enhance the parsimony of the structural model. PLS-SEM is particularly effective in addressing complex models and is well-suited for studies with small sample sizes, non-normal data, or formative indicators, where predictive accuracy is prioritized over theory validation^119,123^. Compared to traditional Structural Equation Modeling (SEM) and experimental approaches, PLS-SEM excels in managing intricate model structures^125,126^. This method offers significant value in application-oriented research and data-constrained contexts, striking a balance between methodological rigor and practical applicability^127,128^. Descriptive statistics, including participant demographics, were computed using SPSS. Hypothesis testing was conducted using the PLS algorithm, with enhanced significance testing through bootstrapping (5000 subsamples), following the guidelines set by Hair et al.^129,130^.

## Results

### Profile of respondents

The demographic breakdown of the study’s respondents revealed a predominance of female tourists, constituting 70% of the sample. A significant portion of the respondents fell into the younger age bracket, with 60.8% aged between 18 and 30 years. The educational level was mainly at the bachelor’s degree level, representing 60.2% of the respondents. Regarding monthly income, 38.1% reported earning less than 3000 RMB, while 22.4% had incomes exceeding 9000 RMB. Visit frequency to the cities under study was high, with 55.5% of respondents having visited more than three times. Duration of stay showed a spread, with 33.3% staying for 3–7 days and 33.6% for more than 14 days. A substantial majority, 74.2%, preferred traveling in groups with family or friends. Detailed demographic information is presented in Table 1.

**Table 1:** Profile of respondents.

Sample (*N* = 357)		
Characteristics		Percentage (%)
Gender	Female	70.0
	Male	30.0
Age	Below 18 years	0.08
	18–30 years	60.8
	31–40 years	23.2
	41–50 years	9.8
	More than 50 years	5.3
Education	High school and below	4.5
	Diploma	10.6
	Bachelor’s degree	60.2
	Master’s degree	20.4
	Doctoral degree	4.2
Occupation	Student	40.6
	Government and public institution employee	21.0
	Self-employed person	1.7
	Freelancer	12.6
	Other	24.1
Personal Monthly Income (RMB)	Less than 3000	38.1
	3000–6000	21.6
	6001–9000	17.9
	More than 9000	22.4
Frequency of Visits to the City	First time	24.6
	Second time	14.8
	Third time	5.0
	More than three times	55.5
Duration of Stay	Less than 3 days	25.8
	3–7 days	33.3
	7–14 days	7.3
	More than 14 days	33.6
Type of Travel Companion	Independent travel	15.7
	Group travel with family/friends	74.2
	Group package travel	3.4
	Others	6.7

### Evaluation of the measurement model and discriminant validity

In accordance with Anderson and Gerbing’s^131^ two-phase approach, this study first validated the measurement model through assessments of convergent validity and reliability. The key criteria for model validation were met: (1) All factor loadings exceeded 0.5, confirming the relevance of the indicators^132^; (2) Composite reliability (CR) scores ranged from 0.7 to 0.95, indicating optimal internal consistency^133^; (3) Average variance extracted (AVE) values exceeded 0.5, demonstrating sufficient variance capture^134^; and (4) All CR measures remained above the conservative threshold of 0.6, ensuring robustness. Collectively, these results affirmed the psychometric strength of the model. Discriminant validity was established using the heterotrait-monotrait ratio (HTMT) method^134^. All inter-construct correlations were below 0.90 (liberal threshold)^135^, with a more stringent analysis revealing values under 0.85—exceeding contemporary methodological standards^136^. This dual-threshold analysis confirmed clear construct differentiation. Comprehensive validity testing across both phases ensured measurement precision and theoretical distinctiveness. Detailed metrics are provided in Tables 2 and 3.

**Table 2:** Measurement model evaluation using PLS.

1st-Order Construct	2nd-Order Construct	Items	Loading	Alpha	CR	AVE
DP1		Wellmanered1	0.849	0.938	0.948	0.697
		Wellmanered2	0.854			
		Wellmanered3	0.828			
		Wellmanered4	0.763			
		Wellmanered5	0.858			
		Wellmanered6	0.879			
		Wellmanered7	0.778			
		Wellmanered8	0.863			
DP2		Vibrancy1	0.890	0.906	0.934	0.781
		Vibrancy2	0.911			
		Vibrancy3	0.919			
		Vibrancy4	0.811			
DP3		Creativity1	0.913	0.922	0.945	0.812
		Creativity2	0.925			
		Creativity3	0.859			
		Creativity4	0.905			
DP4		Conformity1	0.909	0.780	0.869	0.692
		Conformity2	0.893			
		Conformity3	0.673			
DP5		Viciousness1	0.961	0.977	0.983	0.936
		Viciousness2	0.974			
		Viciousness3	0.978			
		Viciousness4	0.956			
ASC		ASC1	0.954	0.908	0.956	0.916
		ASC2	0.960			
ISC		ISC1	0.958	0.906	0.955	0.914
		ISC2	0.954			
SSC		SSC1	0.981	0.963	0.982	0.965
		SSC2	0.983			
ISSC		ISSC1	0.980	0.958	0.980	0.960
		ISSC2	0.980			
FC1		FCamenties1	0.834	0.831	0.888	0.664
		FCamenties2	0.841			
		FCamenties3	0.827			
		FCamenties4	0.756			
FC2		FCattraction1	0.834	0.912	0.932	0.697
		FCattraction2	0.859			
		FCattraction3	0.820			
		FCattraction4	0.901			
		FCattraction5	0.827			
		FCattraction6	0.760			
FC3		FCassessibility1	0.795	0.897	0.921	0.660
		FCassessibility2	0.837			
		FCassessibility3	0.836			
		FCassessibility4	0.815			
		FCassessibility5	0.807			
		FCassessibility6	0.781			
TE1		Joy1	0.943	0.966	0.973	0.879
		Joy2	0.949			
		Joy3	0.953			
		Joy4	0.918			
		Joy5	0.926			
TE2		Love1	0.926	0.950	0.962	0.833
		Love2	0.924			
		Love3	0.904			
		Love4	0.919			
		Love5	0.890			
TE3		PS1	0.900	0.944	0.957	0.816
		PS2	0.908			
		PS3	0.927			
		PS4	0.918			
		PS5	0.981			
	DP	DP1	0.859	0.872	0.913	0.724
		DP2	0.856			
		DP3	0.892			
		DP4	0.793			
	SC	ASC	0.890	0.934	0.953	0.836
		ISC	0.934			
		SSC	0.911			
		ISSC	0.922			
	FC	FC1	0.859	0.879	0.925	0.806
		FC2	0.915			
		FC3	0.917			
	TE	Joy	0.924	0.930	0.955	0.877
		Love	0.963			
		PS	0.922			
PWM		PWM1	0.891	0.913	0.939	0.795
		PWM2	0.929			
		PWM3	0.823			
		PWM4	0.920			

**Table 3:** Discriminant validity via the Heterotrait-Monotrait ratio (HTMT).

	DP	FS	PWOM	SC	TE
DP					
FC	0.717				
PWOM	0.693	0.763			
SC	0.660	0.681	0.622		
TE	0.790	0.869	0.816	0.701	

### Evaluation of the structural model and hypothesis testing

Following the confirmation of the measurement model’s reliability and validity, the structural model was evaluated to test the suggested hypotheses. Employing the methodology advocated by Hair et al.^130^, we examined path coefficients, standard deviations, T-values, P-values, and confidence intervals using bootstrapping techniques. This analysis substantiated the hypothesized relationships among destination personality, self-congruity, functional congruity, and positive word of mouth. Notably, destination personality was found to exert a positive influence on both self-congruity and functional congruity, supporting Hypotheses 1 and 2. Further, self-congruity positively correlated with functional congruity and positive word of mouth, confirming Hypotheses 3 and 4. Functional congruity also demonstrated a significant positive relationship with positive word of mouth, affirming Hypothesis 5. In terms of moderation effects, the interaction between tourists’ emotional experiences and self-congruity significantly impacted positive word of mouth, thus supporting Hypothesis 6. Nevertheless, the expected moderating role of emotions in the link between functional congruity and positive word of mouth did not materialize, resulting in Hypothesis 7 being dismissed. Comprehensive results are depicted in Table 4.

**Table 4:** Evaluation of the structural model.

Path	Beta Value	SD	CIBC (LL)	CIBC (LU)	T value	*P* Value	Supported or Not
H1 DP → SC	0.597	0.041	0.516	0.651	14.672	0.000	Supported
H2 DP → FC	0.409	0.050	0.320	0.490	8.128	0.000	Supported
H3 SC → FC	0.375	0.050	0.284	0.456	7.500	0.000	Supported
H4 SC →PWOM	0.124	0.064	0.028	0.239	1.918	0.028	Supported
H5 FC →PWOM	0.184	0.065	0.078	0.285	2.836	0.002	Supported
H6 TE*SC →PWOM	0.094	0.054	0.025	0.213	1.733	0.042	Supported
H7 TE*FC →PWOM	−0.090	0.071	−0.217	0.010	1.264	0.103	Not

## Discussion and conclusions

Empirical studies within the context of food tourism are relatively scarce. This research enriches the burgeoning field of food tourism by presenting a new, holistic model that integrates self-congruity and functional congruity, aiming to elucidate the connections between destination personality, tourists’ emotional experiences, and positive word of mouth. While previous studies have occasionally applied self-congruity and functional congruity to investigate their impact on tourists’ behavioral intentions, their combined application within the specific domain of food tourism remains rare. Furthermore, this research builds on existing literature that highlights the critical nexus among experiences, emotions, and behaviors^137,138^, by exploring the role of emotional experiences as a moderating variable affecting behavioral intentions. By examining this underexplored intersection, this study advances the theoretical understanding of the dynamics of food tourism and provides valuable insights for destination marketers. These findings can help increase visitor engagement and advocacy by strategically aligning marketing messages with travelers’ functional expectations and self-perceptions.

Unlike most previous studies, this research focused on the four dimensions of self-congruity, revealing the causal relationship between destination personality and self-congruity. Travel destinations fulfill both symbolic and functional roles, meeting travelers’ needs and influencing their behavioral intentions^40,62^. Integrating self-congruity and functional congruity within a unified model of food tourist behavior enhances our understanding of their predictive power on consumer behavior, as suggested by Sirgy et al.^7,92^. This study incorporates these variables into a model that explores self-congruity’s biasing effect on functional congruity and examines their combined influence on tourists’ behavior in food tourism.

Additionally, the study confirmed the significant link between tourists’ emotional experiences and word of mouth behavioral intentions. The results show that tourists’ emotional experiences play a moderating role in the relationship between self-congruity and positive word of mouth. This finding supports the claim by Yang et al.^17^ on the importance of moderating variables in interpreting destination behaviors, adding to the discourse on congruity theories in tourism. However, contrary to expectations, no moderating effect of tourists’ emotional experiences was observed between functional congruity and positive word of mouth. This suggests that the fulfillment of tourists’ functional expectations may inherently prompt word of mouth behaviors, irrespective of the emotional states elicited during the experience. It implies that satisfaction of functional needs alone can influence behavioral intentions towards word of mouth, highlighting a nuanced understanding of the factors driving tourists’ communicative actions.

### Theoretical contributions

This research elucidates the dynamics of self-congruity, functional congruity, and tourists’ emotional experiences in shaping tourists’ behavioral intentions, thereby enriching the discourse on tourist psychology and behavior. By empirically investigating the interplay between destination personality, self-congruity, functional congruity, tourists’ emotional experiences, and positive word of mouth, this study offers significant theoretical insights. The key theoretical contributions are delineated as follows:

Firstly, this study makes an innovative contribution by integrating tourists’ emotional experiences into the theoretical framework of self-congruity and functional congruity, establishing a direct link between emotional experiences, self-congruity, functional congruity, and word of mouth. While prior research has explored self-congruity to examine the interaction between destination personality and tourists’ behaviors (e.g., revisit intentions, consumption choices), few empirical studies have examined the role of emotions as a moderating factor^139–141^. The findings of this study confirm that tourists’ emotional experiences moderate the effect of self-congruity on word of mouth. This not only bridges the existing gap in understanding the role of self-congruity in influencing word of mouth^34,102^, but also expands the framework of self-congruity theory^116,142^. Moreover, despite the limited focus on emotions in current tourism research^17^, this study clarifies that the three dimensions of positive emotional experiences—joy, love, and positive surprise—serve as facilitators in enhancing the impact of self-congruity on word of mouth.

Secondly, this study enhances the theoretical understanding of destination matching mechanisms within coherence theory by distinguishing the roles of self-congruity and functional congruity. While the influence of self-congruity on tourists’ behavioral intentions has been extensively explored in existing literature^34,82^, the theoretical significance of functional congruity has been historically overlooked. This underappreciation has contributed to a cognitive-affective bias in academic models of tourist behavior, overemphasizing psychological identity while neglecting the material dimensions of functional experiences^140^. The findings of this study demonstrate that self-congruity drives behavior by fostering mental resonance between tourists and destinations, whereas functional congruity sustains behavior through tangible, embodied practices—both of which exert a positive influence on tourist actions. Moreover, beyond the direct impact of functional congruity on tourist behavior, this study explores the indirect pathway through which functional congruity influences word of mouth, thus shedding light on additional, more nuanced mechanisms through which functional congruity shapes tourist behavior.

Thirdly, this study contributes to the discourse on word of mouth behavior in tourism research. While word of mouth has been extensively studied in consumer behavior, empirical research focused specifically on the positive and negative dimensions of word of mouth in tourism remains limited^143,144^. Existing literature suggests that positive word of mouth exerts a more substantial impact than negative word of mouth^145^. By examining the role of positive word of mouth within the self-congruity and functional congruence frameworks, this study highlights the facilitating effects of both self-congruity and functional congruence on positive word of mouth, while also revealing the significant moderating role of tourists’ emotional experiences. These findings deepen our understanding of the multidimensional nature of word of mouth and further clarify the distinct roles played by positive and negative word of mouth.

### Practical implications

For tourism management practitioners, particularly those overseeing food tourism destinations, understanding and leveraging emotional engagement is crucial. This study underscores the importance of integrating emotional elements into marketing strategies to enhance tourist attraction and engagement. Emotionally resonant advertising can significantly boost tourists’ motivation and visitation intentions^142^. Successful examples include emotionally charged slogans used by various destinations, such as the evocation of joy and purity by Leshan Cold Cake and the celebration of communal warmth by Chongqing Hot Pot. Therefore, destination marketers are encouraged to meticulously craft and highlight the unique emotional attributes of their culinary offerings. This can be achieved by creating advertising slogans with positive emotional cues, adopting décor styles that evoke positive emotions, and providing services designed to delight visitors. This study demonstrates the efficacy of leveraging destination congruity and personality to forge emotional connections with tourists. Managers can personalize their services to enhance the alignment between tourists’ self-congruity and the destination, fostering an emotional journey that resonates deeply with the target audience.

Secondly, food tourism destination managers are encouraged to curate themed experiences based on festivals or local specialty products, providing tourists with immersive and unique environments, services, activities or amenities. These experiences not only enhance visitor engagement but also foster unique emotional connections. Beyond traditional food-related events, introducing unexpected experiences can attract tourists and generate more traffic. When designing these experiential activities, it is crucial for managers to strategically allocate resources in alignment with the destination’s culinary attributes and services. Projects or activities should be diverse and layered to maximize participation and enrich the visitor experience. Additionally, optimizing spatial arrangements and designs to ensure food safety and hygiene while promoting a serene and cohesive atmosphere will elevate the overall quality of the experience. Meticulous planning and execution are instrumental in encouraging positive word of mouth endorsements, thereby enhancing the destination’s brand impact. Finally, tourism managers should skillfully use online media to publicize and encourage positive word of mouth communication among tourists.

Thirdly, optimizing self-congruity and functional congruity between tourists and food tourism destination brands is a strategic approach to enhancing the appeal of food tourism brands. Destination managers can enhance identification with the destination by developing and amplifying both tangible and intangible, as well as functional and symbolic benefits associated with the food tourism experience. Specifically, enhancing the functional attributes of food tourism destinations—including infrastructure, transportation access, and the quality of restaurant services—can more effectively satisfy tourists’ culinary expectations, thereby improving the destination’ s functional congruity from the tourists’ perspective. Moreover, focusing on word of mouth evaluations to identify deficiencies facilitates continuous improvement of food tourism programs, creating a virtuous cycle of attracting tourists and improving the visitor experience.

### Limitations and future research directions

The scope of this study was limited to five UNESCO-designated Cities of Food within China, focusing exclusively on domestic Chinese tourists. As a result, the findings primarily reflect the perspectives of inland Chinese residents regarding food tourism, which may not be generalizable to international audiences. The regional and demographic specificity of the sample constrains the broader applicability of the results. Future research should consider examining the experiences of foreign visitors to food cities and expanding the sample size to enhance the generalizability of findings. Furthermore, broadening the selection of food tourism destinations to include a diverse range of countries and regions, and conducting comparative analyses, would provide valuable insights into the global variations and commonalities in food tourism experiences.

Additionally, future studies should aim to dissect the influence of tourists’ emotional experiences on word of mouth and other behavioral intentions with greater specificity. Adopting a categorical approach to distinguish among fundamental affective states—such as happiness, anger, and regret—could significantly enhance our understanding of the nuanced predictive power of emotions on tourist behaviors. Such an approach would enable a more detailed validation of how distinct emotional experiences contribute to varying tourist actions and decisions.

Finally, additional important moderating variables like gender and travel companionship may further complicate the influence of self-congruity and functional congruity on positive word of mouth behavioral intentions. The focus of this study has been on travelers’ positive emotional experiences and positive word of mouth. Therefore, future studies should expand the scope to include a greater variety of word of mouth behavioral intents within the context of culinary tourism. This extension may reveal new consumer behavior predictors, providing a more thorough knowledge of the variables influencing word of mouth in the context of food tourism.

## Electronic supplementary material

Below is the link to the electronic supplementary material.

Supplementary Material 1

## Data Availability

Data is provided within the manuscript or supplementary information files.
