# Supplementary material for: Self-congruity and functional congruity drive positive word-of-mouth in food tourism through moderating effects of emotional experiences
Source: Sci Rep. 2025 Mar 27;15:10560. doi: 10.1038/s41598-025-94046-6 (PMC11950296; doi:10.1038/s41598-025-94046-6)
Supplement: Supplementary file 1 — Supplementary Material 1 [file 41598_2025_94046_MOESM1_ESM.docx]

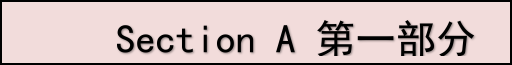


*Introductory Information 介绍性信息*

Kindly mark your response in the most appropriate box.请您在方框中标出您的答案。

1. Which of the following cities or regions have you been to？

您对去过的以下哪座城市或地区印象最深刻呢？（注意：您不能选择您所常住的城市或地区）

□中国四川省成都市 Chengdu, Sichuan Province, China

□中国江苏省扬州市 Yangzhou, Jiangsu Province, China

□中国江苏省淮安市 Huai'an, Jiangsu Province, China

□中国广东省佛山市顺德区 Shunde District, Foshan City, Guangdong Province, China

□中国澳门特别行政区 Macao Special Administrative Region, China

□以上都没有（跳转结束问卷） None of the above (skip to the end of the survey)

2. Have many times have you been to the city? 您去过几次该城市？

□ First time 1次

□ Second time 2次

□ Third time 3次

□ More than three 3次以上

3. How long have you stayed in the city? 您一般在该城市或地区停留多长时间？

□ Less than 3 days 小于3天

□ 3-7 days 3-7天

□ 7-14 days 7-14天

□ More than 14 days 多于14天

4. What types of travel companion do you select in this visitation? 在出行中，您选择的是哪种旅行方式？

□ Independent travel 个人单独出行

□ Group travel with family/friends 与家人朋友一起出行

□ Group package travel 跟团游

□ Others 其他

5.Gender 性别

□ Male男 □ Female女

6.Age 年龄

□ Below 18 years 小于18岁

□ 18-30 years 18-30岁

□ 31-40 years 31-40岁

□ 41-50 years 41-50岁

□ More than 50 years 超过 50 岁

7.Personal monthly income 个人月收入

□ Less than 3000 RMB 少于3000 RMB

□ 3000-6000 RMB

□ 6001-9000 RMB

□ More than 9000 RMB 大于9000 RMB

8. Marital Status 婚姻状况

□ Married 已婚

□ Single 单身

□ Divorced 离婚

□ Other 其他

9. Level of Education 教育背景

□ High school and below 高中及以下

□ Diploma 大专

□ Bachelor’s degree 本科

□ Master’s degree 硕士

□ Doctoral degree 博士

10.Occupation 职业

□ Student 学生

□ Government and public institution employee政府及事业单位员工

□ Self-employed person个体户

□ Freelancer自由职业者

□ Other 其他


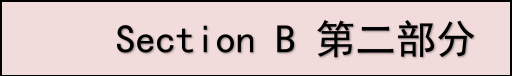


*2.Destination Personality*

*旅游目的地特征*

Listed below are some personality traits that might be associated with the city. We would like you to think of the city as a person. Please indicate to what extent these personality traits accurately describe the city. Check the appropriate box for each personality trait. Please circle your answer. The scales used are interpreted as below. 1=Strongly Disagree (SD), 2=Disagree (D), 3=Somewhat Disagree (SWD), 4=Neither Agree or Disagree (NA/D), 5=Somewhat Agree (SWA), 6=Agree (A), 7=Strongly Agree (SA)

下表展示的是可能适用于描绘被誉为“美食之都”的该城市（您所选择的印象最深刻的城市）性格特征的词汇，如果将该城市视作一个人，您认为以下词汇在多大程度上能够准确描绘该城市？请选出您的答案。评分示意如下：1=完全不同意，2=不同意，3=有些不同意，4=不清楚，5=有些同意，6=同意，7=完全同意。

| Personality Traits associated with the city  与城市相关的个性特点 | Strongly Disagree  完全不同意 | Disagree  不同意 | Somewhat  Disagree  有些不同意 | Neither agree Or Disagree  不清楚 | Somewhat Agree  有些同意 | Agree  同意 | Strongly Agree  完全同意 |
| --- | --- | --- | --- | --- | --- | --- | --- |
| Well-mannered（城市礼仪） | | | | | | | |
| Agreeable  令人愉快的；易相处的 | 1 | 2 | 3 | 4 | 5 | 6 | 7 |
| Sincere  真诚的 | 1 | 2 | 3 | 4 | 5 | 6 | 7 |
| Welcoming  热情好客的 | 1 | 2 | 3 | 4 | 5 | 6 | 7 |
| Peaceful  和平的 | 1 | 2 | 3 | 4 | 5 | 6 | 7 |
| Good  好的 | 1 | 2 | 3 | 4 | 5 | 6 | 7 |
| Respectful  让人尊重的 | 1 | 2 | 3 | 4 | 5 | 6 | 7 |
| Down to earth  淳朴的 | 1 | 2 | 3 | 4 | 5 | 6 | 7 |
| Polite  有礼貌的 | 1 | 2 | 3 | 4 | 5 | 6 | 7 |
| Vibrancy（城市活力） | | | | | | | |
| Diverse  多样化的 | 1 | 2 | 3 | 4 | 5 | 6 | 7 |
| Alive  鲜活的 | 1 | 2 | 3 | 4 | 5 | 6 | 7 |
| Vibrant  充满活力的 | 1 | 2 | 3 | 4 | 5 | 6 | 7 |
| Adventurous  勇于冒险的 | 1 | 2 | 3 | 4 | 5 | 6 | 7 |
| Creativity（城市创造力） | | | | | | | |
| Passionate  有激情的 | 1 | 2 | 3 | 4 | 5 | 6 | 7 |
| Energetic  精力旺盛的 | 1 | 2 | 3 | 4 | 5 | 6 | 7 |
| Enthusiastic  热心的 | 1 | 2 | 3 | 4 | 5 | 6 | 7 |
| Exciting  激动的 | 1 | 2 | 3 | 4 | 5 | 6 | 7 |
| Conformity（城市认同） | | | | | | | |
| Religious  虔诚的 | 1 | 2 | 3 | 4 | 5 | 6 | 7 |
| Spiritual  有灵性的 | 1 | 2 | 3 | 4 | 5 | 6 | 7 |
| Traditional  传统的 | 1 | 2 | 3 | 4 | 5 | 6 | 7 |
| Viciousness（城市黑暗） | | | | | | | |
| Violent  暴力的 | 1 | 2 | 3 | 4 | 5 | 6 | 7 |
| Depraved  颓废的 | 1 | 2 | 3 | 4 | 5 | 6 | 7 |
| Obscure  隐晦的 | 1 | 2 | 3 | 4 | 5 | 6 | 7 |
| Vulgar  粗俗的 | 1 | 2 | 3 | 4 | 5 | 6 | 7 |


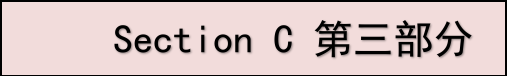


*3.Self-congruity*

*自我一致性*

First, please think about the city as a person and think about the personality characteristics of the city. Next, think about how you see yourself and how you would like to see yourself. Then, indicate if there are any differences between you and the city in terms of personality characteristics by indicating your agreement or disagreement to the following statements using the scale below: 1=Strongly Disagree (SD), 2=Disagree (D), 3=Neutral (N), 4=Agree (A), 5=Strongly Agree (SA)

首先请您将被誉为“美食之都”的该城市（您所选择的印象最深刻的城市）视作一个人，并思考一下与该城市相关的一些个性特点。然后请您思考一下在现实生活中，您是怎么看待自身的？在理想情况中，您觉得自己应该是什么样的？就个性特点而言，您在多大程度上认同这些关于该城市与您自身个性异同点的描述？请选出您的答案。评分示意如下：1=完全不同意，2=不同意，3=中立，4=同意，5=完全同意。

|  | Strongly Disagree  完全不同意 | Disagree  不同意 | Neutral  中立 | Agree  同意 | Strongly Agree  完全同意 |
| --- | --- | --- | --- | --- | --- |
| Actual self-congruity 实际的自我一致性 | | | | | |
| The image of a typical resident of the city is similar to who I am.  该美食之都的典型居民形象和我很相似。 | 1 | 2 | 3 | 4 | 5 |
| The image of a typical resident of the city is similar to how I see myself.  该美食之都的典型居民形象与我对自己的看法很相似。 | 1 | 2 | 3 | 4 | 5 |
| Ideal self-congruity 理想的自我一致性 | | | | | |
| The image of a typical resident of the city is similar to who I would like to be.  该美食之都的典型居民形象和我想成为的人是相似的。 | 1 | 2 | 3 | 4 | 5 |
| The image of a typical resident of the city is similar to how I would like to see myself.  该美食之都的典型居民形象与我想看到的自己是相似的。 | 1 | 2 | 3 | 4 | 5 |
| Social self-congruity 社会的自我一致性 | | | | | |
| The image of a typical resident of the city is similar to who others believe I am.  该美食之都的典型居民形象和别人认为的我很相似。 | 1 | 2 | 3 | 4 | 5 |
| The image of a typical resident of the city is similar to how others see me.  该美食之都的典型居民形象与其他人对我的看法很相似。 | 1 | 2 | 3 | 4 | 5 |
| Ideal social self-congruity 理想的社会自我一致性 | | | | | |
| The image of a typical resident of the city is similar to how I would like others to see me.  该美食之都的典型居民形象与我希望别人看到的我的形象相似。 | 1 | 2 | 3 | 4 | 5 |
| The image of a typical resident of the city is similar to how I ideally like to be seen by others.  该美食之都的典型居民形象与我理想中喜爱被别人看到的样子相似。 | 1 | 2 | 3 | 4 | 5 |


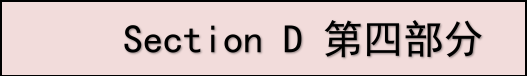


*4.Functional-congruity*

How satisfied are you with the city you selected as the most impressive as a travel destination in the following aspects? Please select your rating. The scoring guide is as follows: 1 = Much worse than I expected, 2 = Worse than I expected, 3 = Neutral, 4 = Better than I expected, 5 = Much better than I expected.

您在以下方面对被誉为“美食之都”的该城市（您所选择的印象最深刻的城市）作为旅游目的地的满意度如何？请选出来。评分示意如下：1=比我预期差得很多，2=比我预期差，3=中立，4=和我预期好，5=比我预期好很多。

|  | Much worse than I expected  比我预期差很多 | Worse than I expected  比我预期差 | Neutral  中立 | Better than I expected  比我预期好 | Much better  than I expected  比我预期好很多 |
| --- | --- | --- | --- | --- | --- |
| Amenities 设施 | | | | | |
| Quality of restaurants  餐馆的质量 | 1 | 2 | 3 | 4 | 5 |
| Value for money spent  物有所值 | 1 | 2 | 3 | 4 | 5 |
| Local cuisine  当地的美食 | 1 | 2 | 3 | 4 | 5 |
| Attractions 吸引点 | | | | | |
| Quality of accommodations  环境质量 | 1 | 2 | 3 | 4 | 5 |
| Cultural heritage  文化遗产 | 1 | 2 | 3 | 4 | 5 |
| Historical attractions  历史景点 | 1 | 2 | 3 | 4 | 5 |
| Local tourist/excursions  当地旅行 | 1 | 2 | 3 | 4 | 5 |
| Cultural attractions  文化景点 | 1 | 2 | 3 | 4 | 5 |
| Accessibility and tourist friendliness 无障碍设施和旅游友好性 | | | | | |
| Variety of tourist activities  丰富的旅游活动 | 1 | 2 | 3 | 4 | 5 |
| Availability of local festivals  当地活动／节日的易参与性 | 1 | 2 | 3 | 4 | 5 |
| Quality of public transportation  公共交通的质量 | 1 | 2 | 3 | 4 | 5 |
| Accessibility of the destination  目的地可达性 | 1 | 2 | 3 | 4 | 5 |
| Traffic infrastructure  交通基础设施 | 1 | 2 | 3 | 4 | 5 |
| Local people’s hospitality  当地人的热情好客度 | 1 | 2 | 3 | 4 | 5 |
| Attitude of staff in tourism旅游工作人员的态度 | 1 | 2 | 3 | 4 | 5 |
| Safety and security  安全性 | 1 | 2 | 3 | 4 | 5 |


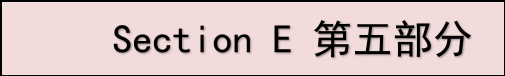


1. *Emotions*

To what extent do you agree with the following emotional descriptions about visiting the city you selected as the most impressive? Please choose your answer. The scoring guide is as follows: 1 = Strongly disagree, 2 = Disagree, 3 = Somewhat disagree, 4 = Neither agree or Disagree, 5 = Somewhat agree, 6 = Agree, 7 = Strongly agree.

针对以下参观该“美食之都”城市（您所选择的印象最深刻的城市）的情绪描述，您在多大程度上认同这些描述？请选出您的答案。评分示意如下：1=完全不同意，2=不同意，3=有些不同意，4=不清楚，5=有些同意，6=同意，7=完全同意。

| Emotions  情绪 | Strongly Disagree  完全不同意 | Disagree  不同意 | Somewhat  Disagree  有些不同意 | Neither agree or Disagree  不清楚 | Somewhat Agree  有些同意 | Agree  同意 | Strongly Agree  完全同意 |
| --- | --- | --- | --- | --- | --- | --- | --- |
| Joy | | | | | | | |
| Visiting the city I feel pleasure  参观该美食之都我感到愉悦 | 1 | 2 | 3 | 4 | 5 | 6 | 7 |
| Visiting the city I feel joy  参观该美食之都我感到开心 | 1 | 2 | 3 | 4 | 5 | 6 | 7 |
| Visiting the city I feel delight  参观该美食之都我感到快乐 | 1 | 2 | 3 | 4 | 5 | 6 | 7 |
| Visiting the city I feel cheerfulness  参观该美食之都我感到欣喜 | 1 | 2 | 3 | 4 | 5 | 6 | 7 |
| Visiting the city I feel enthusiasm  参观该美食之都我感到充满热情 | 1 | 2 | 3 | 4 | 5 | 6 | 7 |
| Love | | | | | | | |
| Visiting the city I feel warmth  参观该美食之都我感到温暖 | 1 | 2 | 3 | 4 | 5 | 6 | 7 |
| Visiting the city I feel tenderness  参观该美食之都我感到亲切 | 1 | 2 | 3 | 4 | 5 | 6 | 7 |
| Visiting the city I feel affection  参观该美食之都我感到很喜爱 | 1 | 2 | 3 | 4 | 5 | 6 | 7 |
| Visiting the city I feel love  参观该美食之都我感到对它的爱意 | 1 | 2 | 3 | 4 | 5 | 6 | 7 |
| Visiting the city I feel caring  参观该美食之都我感到对它的关切 | 1 | 2 | 3 | 4 | 5 | 6 | 7 |
| Positive surprise | | | | | | | |
| Visiting the city I feel inspiration  参观该美食之都我充满了灵感 | 1 | 2 | 3 | 4 | 5 | 6 | 7 |
| Visiting the city I feel fascinated  参观该美食之都我备受吸引 | 1 | 2 | 3 | 4 | 5 | 6 | 7 |
| Visiting the city I feel astonished  参观该美食之都我感到惊奇 | 1 | 2 | 3 | 4 | 5 | 6 | 7 |
| Visiting the city I feel surprised  参观该美食之都我感到惊喜 | 1 | 2 | 3 | 4 | 5 | 6 | 7 |
| Visiting the city I feel amazed  参观该美食之都我感到惊叹 | 1 | 2 | 3 | 4 | 5 | 6 | 7 |
| Unpleasantness | | | | | | | |
| Visiting the city I feel unhappiness  参观该美食之都我感到不开心 | 1 | 2 | 3 | 4 | 5 | 6 | 7 |
| Visiting the city I feel regret  参观该美食之都我感到后悔 | 1 | 2 | 3 | 4 | 5 | 6 | 7 |
| Visiting the city I feel disappointment  参观该美食之都我感到失望 | 1 | 2 | 3 | 4 | 5 | 6 | 7 |
| Visiting the city I feel sadness  参观该美食之都我感到难过 | 1 | 2 | 3 | 4 | 5 | 6 | 7 |
| Visiting the city I feel displeasure  参观该美食之都我感到不快乐 | 1 | 2 | 3 | 4 | 5 | 6 | 7 |
| Note: The degree of displeasure is not as strong as unhappiness.注：displeasure程度没有unhappiness强。 | | | | | | | |


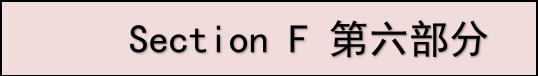


*6.Word of Mouth*

As a visitor to the city known you selected as the most impressive, to what extent do you agree with the following descriptions? Please select your answer. The scoring guide is as follows: 1 = Strongly disagree, 2 = Disagree, 3 = Neutral, 4 = Agree, 5 = Strongly agree.

您作为去过被誉为“美食之都”的该城市（您所选择的印象最深刻的城市）的游客，以下描述您在多大程度上赞同？请选出您的答案。评分示意如下：1=完全不同意，2=不同意，3=中立，4=同意，5=完全同意。

|  | Strongly Disagree  完全不同意 | Disagree  不同意 | Neutral  中立 | Agree  同意 | Strongly Agree  完全同意 |
| --- | --- | --- | --- | --- | --- |
| Positive WOM 正面口碑 | | | | | |
| I will say positive things about the city to other people.  我会对其他人说一些关于这个被誉为“美食之都”城市的正面看法。 | 1 | 2 | 3 | 4 | 5 |
| I will recommend the city to someone who seeks my advice.  我会向寻求我建议的人推荐这个被誉为“美食之都”的城市。 | 1 | 2 | 3 | 4 | 5 |
| I will encourage friends and relatives to stay at the city.  我会鼓励朋友和亲戚留在这个被誉为“美食之都”的城市。 | 1 | 2 | 3 | 4 | 5 |
| I’m likely to spread positive word-of-mouth about the city.  我很可能会传播关于这个“美食之都”城市的正面口碑。 | 1 | 2 | 3 | 4 | 5 |
| Negative WOM 负面口碑 | | | | | |
| I would complain about the city to other people.  我会向别人抱怨这个被誉为“美食之都”的城市。 | 1 | 2 | 3 | 4 | 5 |
| I would say negative things about the city to other people.  我会对别人说一些关于有“美食之都”称号的该城市的负面事情。 | 1 | 2 | 3 | 4 | 5 |
| I would recommend other people not to book flights to the city.  我会建议其他人不要预订去这个有“美食之都”称号的城市的航班。 | 1 | 2 | 3 | 4 | 5 |
